# Effect of perioperative goal-directed fluid therapy on postoperative complications after thoracic surgery with one-lung ventilation: a systematic review and meta-analysis

**DOI:** 10.1186/s12957-023-03169-5

**Published:** 2023-09-18

**Authors:** Xuan Li, Qinyu Zhang, Yuyang Zhu, Yihan Yang, Wenxia Xu, Yufei Zhao, Yuan Liu, Wenqiang Xue, Yu Fang, Jie Huang

**Affiliations:** Department of Anesthesiology, The First Affiliated Hospital of Kunming Medical University, KunMing, China

**Keywords:** Goal-directed fluid therapy, Meta-analysis, One-lung ventilation, Postoperative complications, Pulmonary protection

## Abstract

**Background:**

An understanding of the impact of goal-directed fluid therapy (GDFT) on the outcomes of patients undergoing one-lung ventilation (OLV) for thoracic surgery remains incomplete and controversial. This meta-analysis aimed to assess the effect of GDFT compared to other fluid therapy strategies on the incidence of postoperative complications in patients with OLV.

**Methods:**

The Embase, Cochrane Library, Web of Science, and MEDLINE via PubMed databases were searched from their inception to November 30, 2022. Forest plots were constructed to present the results of the meta-analysis. The quality of the included studies was evaluated using the Cochrane Collaboration tool and Risk Of Bias In Non-Randomized Study of Interventions (ROBINS-I). The primary outcome was the incidence of postoperative complications. Secondary outcomes were the length of hospital stay, PaO_2_/FiO_2_ ratio, total fluid infusion, inflammatory factors (TNF-α, IL-6), and postoperative bowel function recovery time.

**Results:**

A total of 1318 patients from 11 studies were included in this review. The GDFT group had a lower incidence of postoperative complications [odds ratio (OR), 0.47; 95% confidence interval (95% CI), 0.29–0.75; *P* = 0.002; *I* ^2^, 67%], postoperative pulmonary complications (OR 0.48, 95% CI 0.27–0.83; *P* = 0.009), and postoperative anastomotic leakage (OR 0.51, 95% CI 0.27–0.97; *P* = 0.04). The GDFT strategy reduces total fluid infusion.

**Conclusions:**

GDFT is associated with lower postoperative complications and better survival outcomes after thoracic surgery for OLV.

**Supplementary Information:**

The online version contains supplementary material available at 10.1186/s12957-023-03169-5.

## Introduction

The average cost of a lobectomy is estimated at $6549, consisting mostly of increased length of hospital stay (LOS) due to postoperative complications [1]. Additionally, serious complications remain a significant cause of postoperative mortality. Fluid overload is associated primarily with pulmonary complications in this patient population [2, 3], while hypovolemia may cause surgical site necrosis, infection, and damage to the newly constructed anastomosis [4]. Goal-directed fluid therapy (GDFT) aims to achieve an appropriate balance between fluid overload and hypovolemia [4].

Perioperative fluid therapy is one of the most controversial topics in anesthesia practice [5]. Many clinicians use pulse rate, blood pressure, central venous pressure (CVP), and urine volume to assess fluid responsiveness. However, such static measurements are poor predictors of fluid responsiveness [6, 7]. According to recent findings, changes in dynamic perioperative hemodynamic parameters [e.g., stroke volume (SV), stroke volume variation (SVV), cardiac index (CI)] appear to be better predictors of fluid responsiveness [8–10]. GDFT involves the assessment of hemodynamic variables and optimization of fluid therapy using a goal-directed approach, which is the cornerstone of tissue perfusion and oxygenation [11]. Rational fluid therapy can reduce the patient’s stress response to surgical trauma, thus improving the quality of perioperative care and patient prognosis [12–14].

In a recent comprehensive meta-analysis, Jessen et al. showed that GDFT could reduce mortality and the risk of several postoperative complications in patients undergoing non-cardiothoracic surgery; however, patients with one-lung ventilation (OLV) were not evaluated [15]. Several randomized controlled trials (RCTs) have shown that GDFT positively reduces inflammatory factors and postoperative complications after lung resection [11, 16, 17]. However, the assessment of organ-specific complications is biased. For instance, improvement in postoperative outcomes with fluid therapy in patients undergoing esophagectomy remains inconclusive [4, 18]. To date, no meta-analysis evaluating the outcomes of perioperative GDFT in patients undergoing thoracic surgery with OLV has been conducted. Therefore, we conducted this systematic review and meta-analysis to comprehensively assess the impact of GDFT compared with other fluid therapies, including conventional fluid therapy (CFT) and restrictive fluid therapy (RFT), on postoperative complications and other prognostic indicators.

## Methods

This study has been reported in line with PRISMA [19]. This meta-analysis has been registered on PROSPERO (https://www.crd.york.ac.uk/prospero/display_record.php?RecordID=388124). Registration number: CRD42023388124.

### Search strategy

Embase, Cochrane Library, Web of Science, and MEDLINE via PubMed databases were independently searched from the first record to November 30, 2022. The keywords searched included medical subject terms (MeSH) related to GDFT. The search results were combined with MeSH terms related to thoracic surgical procedures. Supplementary Table 1 records the search formula and search results for each database. Two authors (XL and QZ) screened all studies after excluding duplicate studies and screened references of included studies for additional relevant studies, and disagreements were resolved by discussion with the third author (YF).

### Inclusion and exclusion criteria

Inclusion and exclusion criteria for this systematic review and meta-analysis were identified according to the PICOS (Patient, Intervention, Comparison, Outcome, Study Design) strategy.

1. Patients: adults (≥ 18 years) who underwent thoracic surgery (esophageal surgery, lung resection) with OLV. Patients who underwent thoracic surgery but were ventilated by double lung ventilation were excluded.
2. Type of intervention: GDFT strategy for perioperative fluid management. That is, trials investigating treatment protocols designed to achieve one or more specific hemodynamic goals (e.g., SVV, SV, CI). There is no restriction on the type of hemodynamic goals, nor is there a restriction on the device used to measure it. Trials targeting blood pressure are traditionally not considered GDFT and were excluded:
3. Type of comparison: The accepted comparator is CFT or other fluid therapy.
4. Type of outcome: Eligible studies must report at least one of the following outcomes: incidence of postoperative complications or perioperative physiological functional parameters of the patient [e.g., PaO_2_/FiO_2_ ratio (P/F ratio), inflammatory factors].
5. Types of studies: RCTs, observational studies with a control group. Reviews, case reports, animal experiments, and observational studies without control groups were excluded.

### Outcome measure

Primary outcome: Postoperative complications. These include postoperative pulmonary complications (PPCs, assessed using the Melbourne Group Scale [20]), postoperative cardiovascular complications (e.g., arrhythmia, myocardial infarction, cardiovascular dysfunction, heart failure, and postoperative myocardial injury), postoperative renal complications(e.g., acute kidney injury (AKI), renal failure, renal dysfunction, and elevation of serum creatinine level by > 50%), postoperative delirium (POD), and anastomotic leakage.

Secondary outcomes: (1) LOS, (2) total fluid infusion, (3) P/F ratio, (4) inflammatory factors, and (5) postoperative bowel function recovery time.

### Data extraction

Two authors (XL and QZ) reviewed individual studies and extracted data using a pre-defined standardized data extraction form. The following data were retrieved: first author, publication year, sample size, type of study, type of surgery, the goals of GDFT, fluid therapy strategy for the control group, and outcomes of the study. Disagreements were resolved by discussion with the third author (YF). The details of included studies are shown in Table 1.

**Table 1:** Characteristics of the included studies

Reference	Country	Study type	Population (*n*)	Surgery	E group	Goal	C group	Outcomes
Wang et al. 2021 [17]	China	RCT	GDFT: *n* = 40 Control: *n* = 34	Lung cancer surgery	GDFT	SV 10%	CFT	Postoperative complications; Fluid balance Hemodynamic indexes; LOS; Inflammatory factors; Recovery time of bowel function.
Veelo et al. 2017 [21]	Netherlands	Observational study	GDFT: *n* = 100 Control: *n* = 99	Esophageal surgery	GDFT	Optimal SV	CFT	Postoperative complications; Mortality; Length of ICU and hospital stay; Fluid balance.
Zhang et al. 2013 [11]	China	RCT	GDFT: *n* = 30 Control: *n* = 30	Thoracoscopy lobectomy	GDFT	SVV 10% ± 1% CI > 2.5 ml min^−1^ m^−2^	CFT	Postoperative complications; P/F ratio; Fluid balance; LOS.
Xu et al. 2017 [16]	China	RCT	GDFT: *n* = 84 Control: *n* = 84	Thoracoscopy lobectomy	GDFT	SVV 10%-13% CI > 2.5 ml min^−1^ m^−2^	CFT	Postoperative complications; P/F ratio; Inflammatory factors; LOS; Respiratory mechanics.
Bahlmann et al. 2019 [4]	Sweden	RCT	GDFT: *n* = 30 Control: *n* = 29	Oesophagectomy	GDFT	Optimal SV CI > 2.5 ml min^−1^ m^−2^ MAP > 65 mmHg	CFT	Postoperative complications; Length of ICU and hospital stay; Mortality; Fluid balance; Recovery time of bowel function.
Mukai et al. 2020 [18]	Japan	RCT	GDFT: *n* = 115 Control: *n* = 117	Oesophagectomy	GDFT	SVV < 8%; SV decrease < 10% BP > 90 mmHg	CFT	Postoperative complications; Mortality; Length of ICU and hospital stay; Recovery time of bowel function.
Sahutoglu et al. 2018 [22]	Turkey	Observational study	GDFT: *n* = 43 Control: *n* = 45	Lobectomy	GDFT	SVV < 13%	CFT	Postoperative complications; Fluid balance.
Kaufmann et al. 2017 [23]	Germany	RCT	GDFT: *n* = 48 Control: *n* = 48	Lung surgery	GDFT	SVV < 10% CI > 2.5 ml min^−1^ m^−2^ MAP > 70 mmHg	CFT	Postoperative complications; LOS; Hemodynamic indexes.
Tang et al. 2021 [24]	China	RCT	GDFT: *n* = 33 Control: *n* = 32	Minimally invasive esophagectomy	GDFT	SVV < 11%	CFT	Postoperative complications; Mortality; LOS; Fluid balance.
Wang et al. 2022 [25]	China	RCT	GDFT: *n* = 78 Control: *n* = 81	Thoracoscopy lobectomy	GDFT	rScO_2_ ± 20% (baseline level)	CFT	Postoperative complications; Inflammatory factors; Awakening time; VAS score.
Li et al. 2021 [26]	China	RCT	GDFT: *n* = 59 Control: *n* = 59	Thoracoscopy lobectomy	GDFT	SVV 10%-13% CI > 2.5 ml min^−1^ m^−2^	RFT	Postoperative complications; Mortality; Length of hospital stay; Fluid balance.

*BP* blood pressure, *C group* control group, *CI* cardiac index, *CFT* conventional fluid therapy, *E group* experimental group, *GDFT* goal-directed fluid therapy, *LOS* length of hospital stay, *MAP* mean arterial pressure, *RCT* randomized controlled trial, *rScO*_*2*_ regional saturation of cerebral oxygenation, *RFT* restrictive fluid therapy, *SV* stroke volume, *SVV* stroke volume variation

### Quality assessment

We independently assessed the methodological quality and risk of bias of RCTs using the Cochrane Collaboration tool. The observational studies were evaluated according to the standard of the Risk of Bias In Non-randomiszed Study of Interventions (ROBINS-I). ROBINS-I evaluates how well the observational study handles bias spread across seven domains compared to a well-performed RCT. The seven domains are (1) confounding, (2) selection of participants, (3) classification of interventions, (4) deviations from intended interventions, (5) missing data, (6) measurement of outcomes, and (7) selection of reported results. Two authors (XL and QZ) extracted data independently to reduce risk and other biases, and disagreements were resolved by discussion with the third author (YF).

### Statistical analysis

Review Manager software (RevMan version 5.4) was used to conduct the meta-analysis. The coefficient *I* ^2^ was calculated to assess heterogeneity, with levels of heterogeneity defined as low (25–49%), medium (50–74%), and high (> 75%) levels. Because of clinical methodological heterogeneity and other potential heterogeneity in the included studies, we used a random-effects model for all data analyses. Whenever significant heterogeneity existed, we searched for possible sources of heterogeneity for the meta-analysis by sensitivity analysis. Owing to differences in the definition of postoperative complications, site of complications, type of surgery, type of study, and specific hemodynamic goals of GDFT among the included studies, we performed further subgroup analyses of the primary outcome. We used the 95% CI for dichotomous variables to calculate the odds ratios (OR), and for continuous variables, we used the mean difference (MD). When reporting continuous variables as medians and ranges in some studies, we used the method described by McGrath et al. to estimate the mean and standard deviation for data pooling for continuous variables [27]. *P* < 0.05 was considered the difference to be statistically significant.

## Results

### Selection of studies and study characteristics

**Fig. 1:**
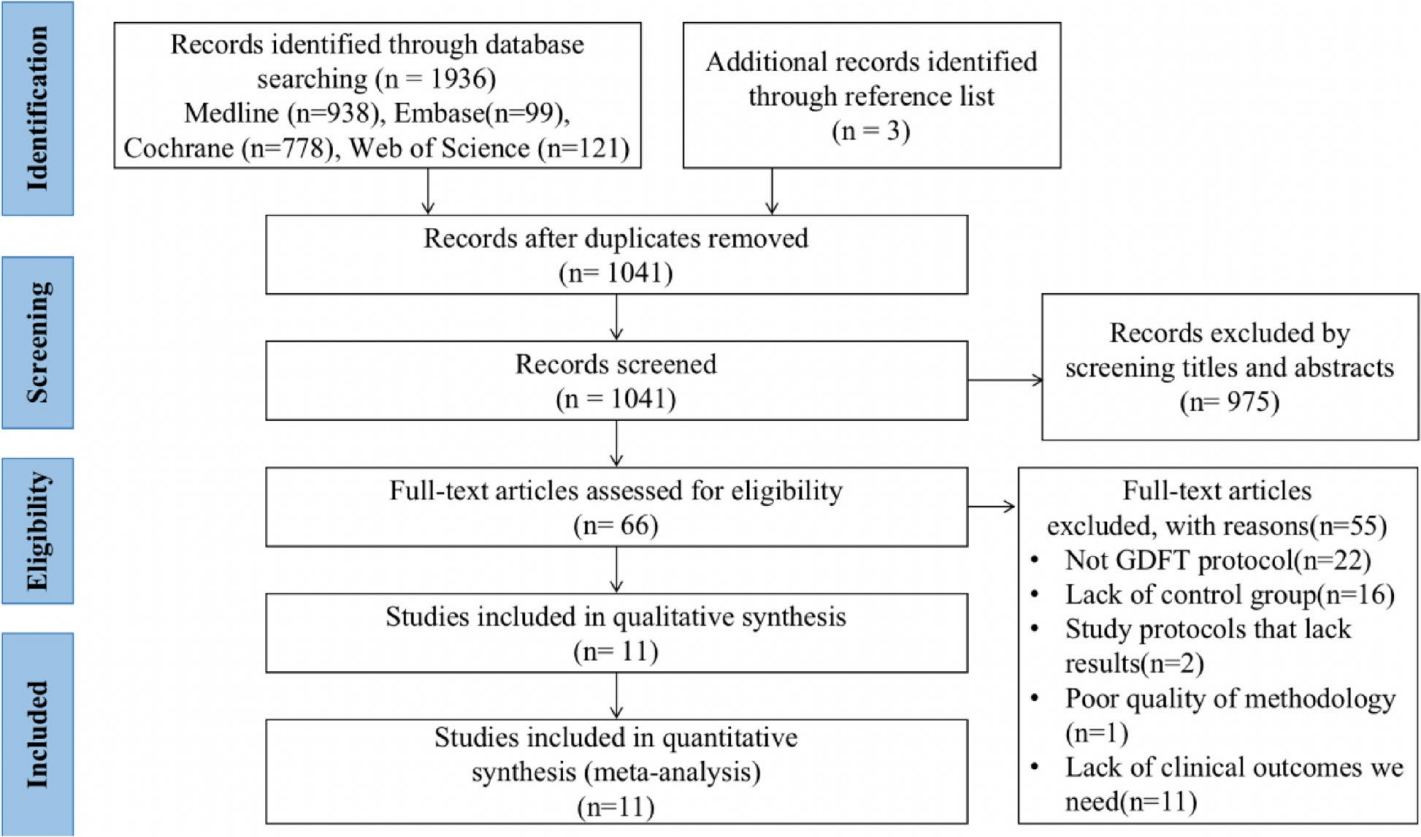
Flow diagram of the study selection

Using the search strategy explained in the previous section, we obtained 1936 relevant studies in our initial search and three additional studies in our manual review of the references. A full-text review of 66 of these studies was conducted, 11 of which were selected [4, 11, 16–18, 21–26]. The screening process is shown in Fig. 1, and Table 1 presents the basic characteristics of the included studies. The sample sizes of the studies ranged from 59 to 232 patients, and a total of 1318 patients were analyzed. Five of the included studies had a sample size of more than 100 patients (45%) [16, 18, 21, 25, 26], nine studies were RCTs [4, 11, 16–18, 23–26], and two were observational studies [21, 22]. Ten studies compared GDFT with CFT [4, 11, 16–18, 21–25], and one study compared GDFT with RFT [26]. For the RFT strategy, fluids and norepinephrine were administered to maintain the mean arterial pressure (MAP) > 65 mmHg [26]. Since MAP as a hemodynamic goal is not traditionally considered GDFT [15], we concluded that two different GDFT protocols were not being compared, and thus, this study was included in our meta-analysis. We conducted subgroup and sensitivity analyses to explore whether this study contributed to heterogeneity.

### Quality assessment

**Fig. 2:**
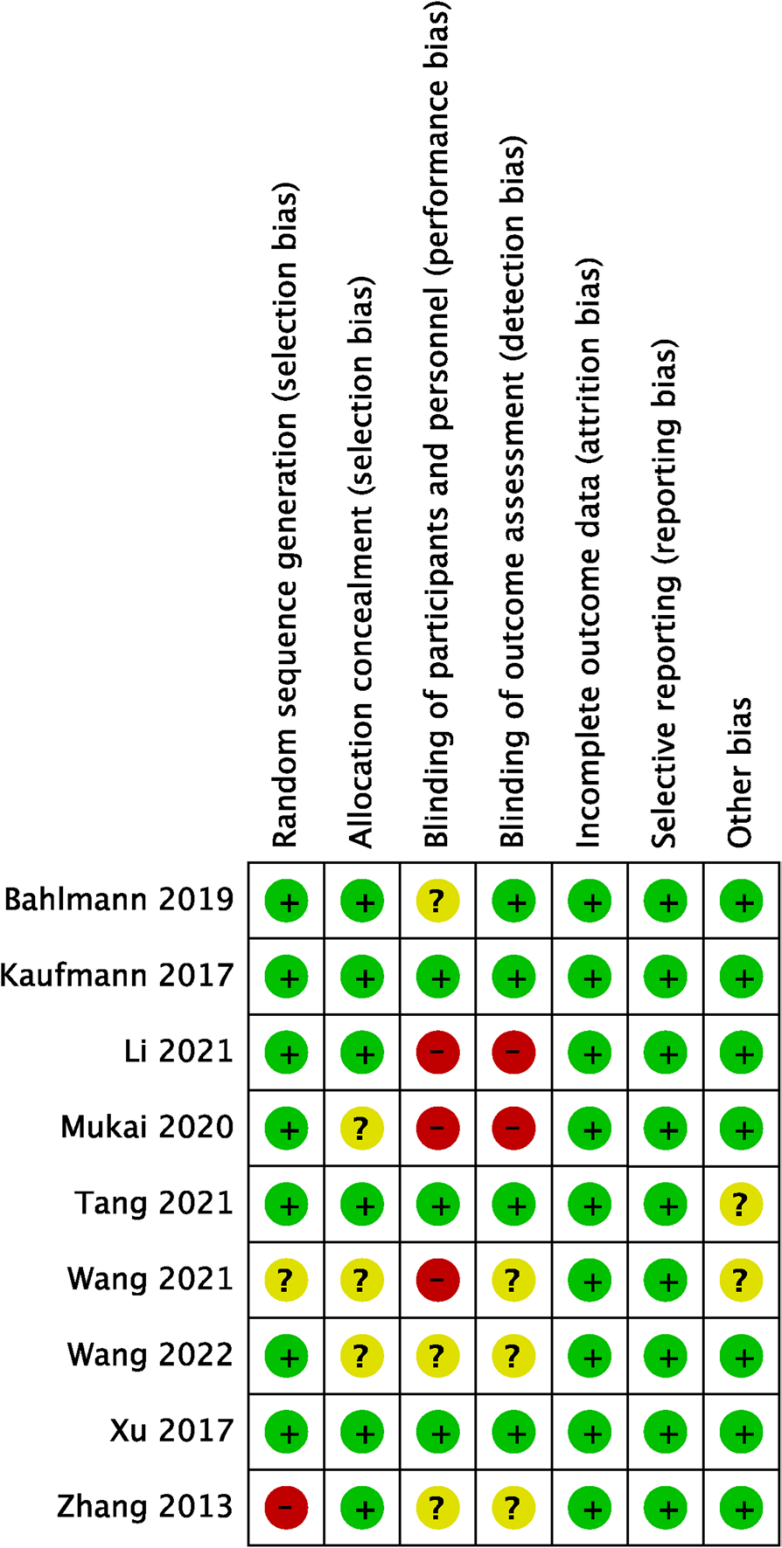
Risk of bias assessment of the included RCTs

**Fig. 3:**
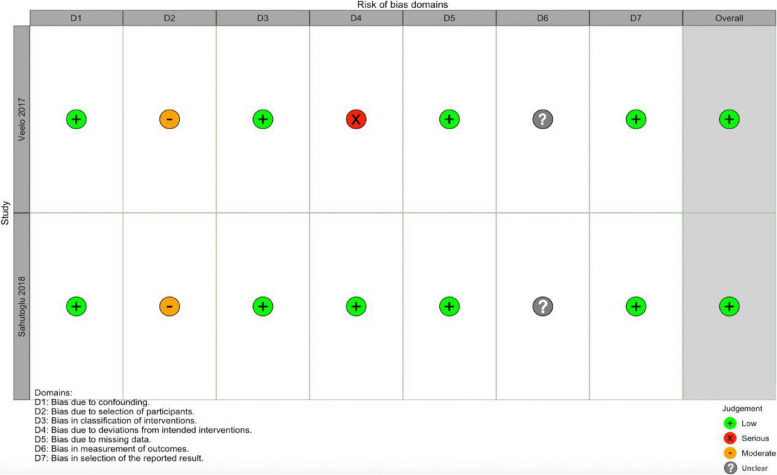
Risk of bias assessment of the included observational studies

Our quality assessment was based on the Cochrane Collaboration tool and ROBINS-I (Figs. 2 and 3). Most RCTs had a low risk of bias, both observational studies had a moderate degree of selection bias [21, 22], and Veelo et al.’s study had a serious degree of bias due to deviations from intended interventions [21]. Overall risk of bias was low for both studies [21, 22].

### Primary outcome

#### Total postoperative complications

**Fig. 4:**
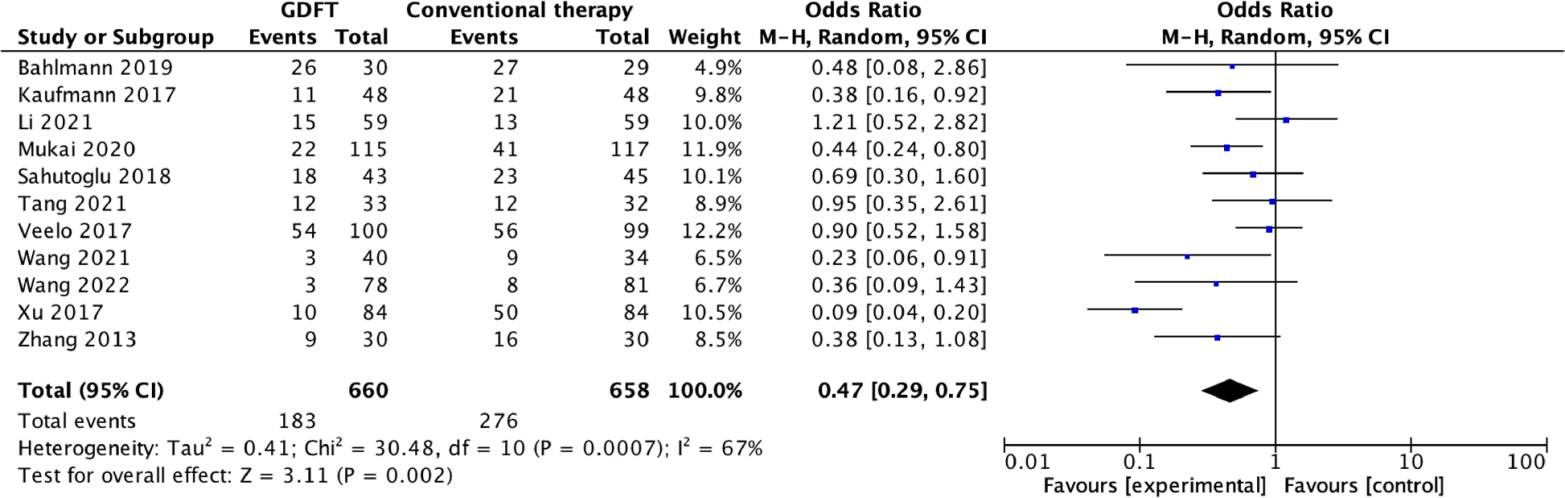
Forest plot of summary data for the number of total patients with postoperative complications

All included studies reported postoperative complications [4, 11, 16–18, 21–26]. The overall incidence of postoperative complications was 183/660 (27.7%) in the GDFT group and 276/658 (41.9%) in the control group. The pooled OR of 0.47 indicated that perioperative GDFT was associated with a reduction in postoperative complications (95% CI 0.29–0.75; *P* = 0.002; *I* ^2^ = 67%) (Fig. 4).

#### Subgroup analyses of postoperative complications

##### Organ-specific complications

**Fig. 5:**
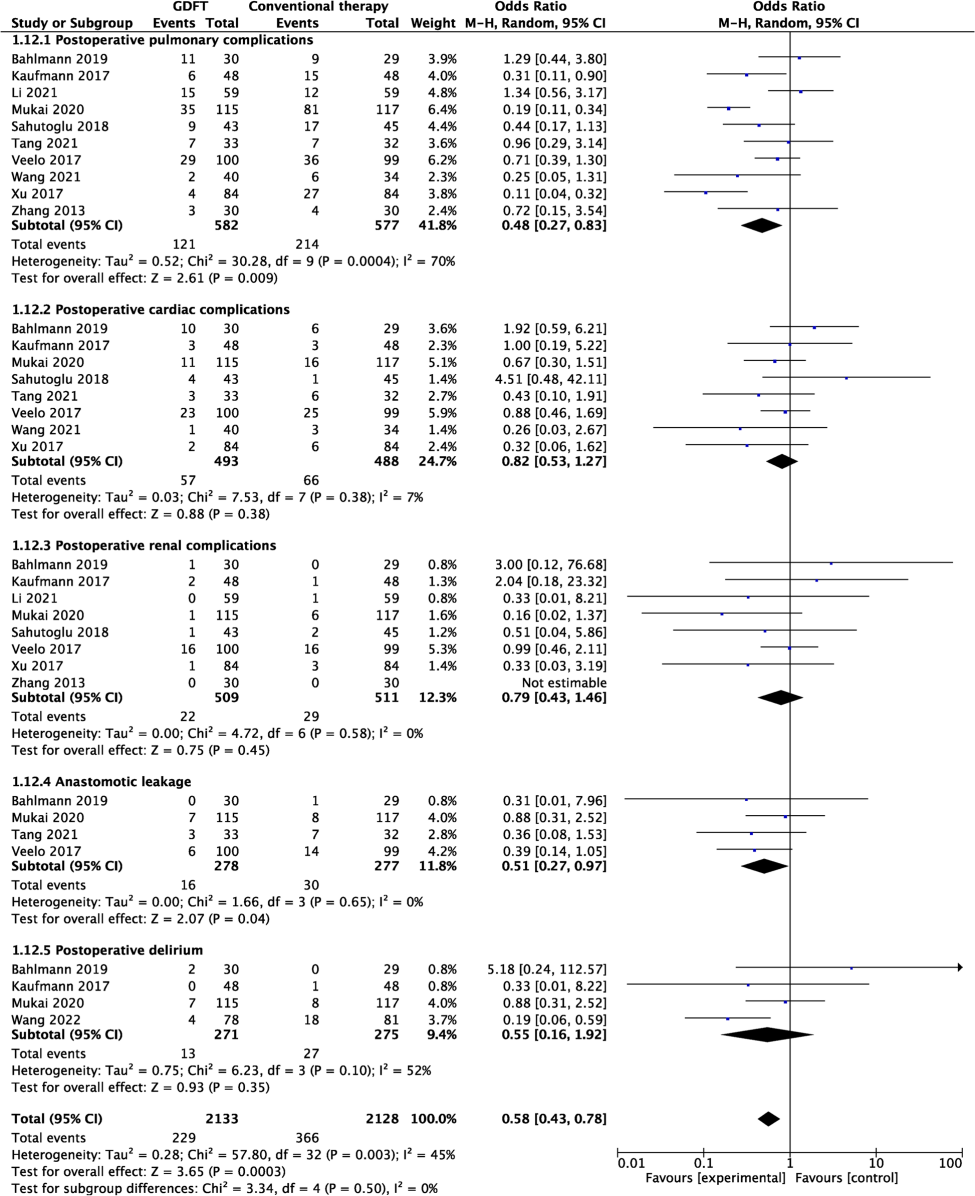
Forest plot of summary data for the number of patients with organ-specific complications

Statistically significant differences were observed in PPCs (OR 0.48, 95% CI 0.27–0.83; *P* = 0.009; *I* ^2^ = 70%) and anastomotic leakage (OR 0.51, 95% CI 0.27–0.97; *P* = 0.04; *I* ^2^ = 0%) between the two groups. However, no statistically significant differences were found for postoperative cardiovascular complications, renal complications, or POD (Fig. 5).

##### Surgical procedures and postoperative complications

**Fig. 6:**
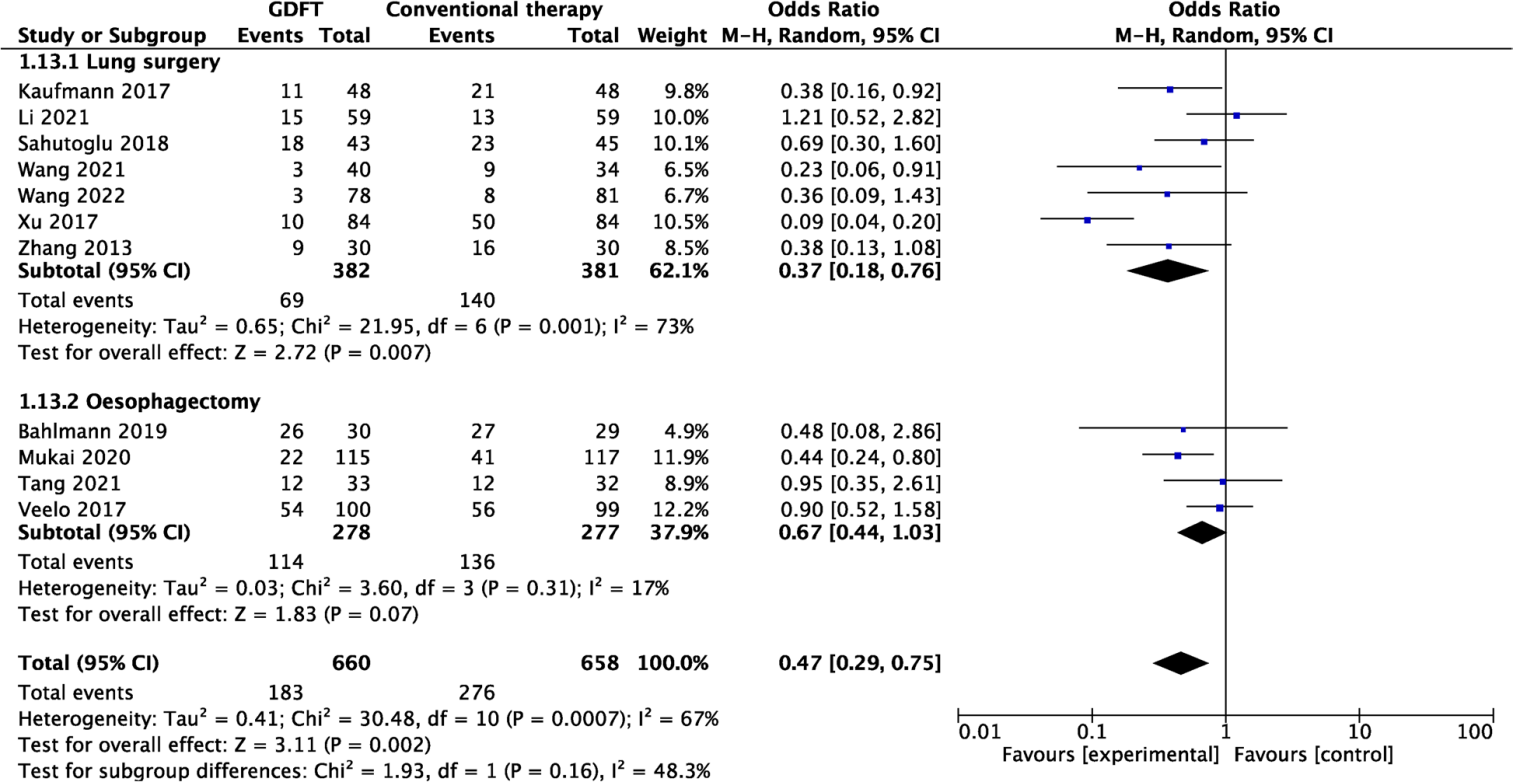
Forest plot comparing postoperative complications for the GDFT versus the conventional therapy according to surgical procedure

For lung resections (7/11) [11, 16, 17, 22, 23, 25, 26], the incidence of postoperative complications was lower in the GDFT group (OR 0.37, 95% CI 0.18–0.76; *P* = 0.007; *I* ^2^ = 73%). However, for esophagectomies, no statistically significant difference in the incidence of postoperative complications between the two groups was found (4/11) [4, 18, 21, 24] (Fig. 6).

##### Hemodynamic goals and postoperative complications

Among the studies that used the SVV and/or CI as goals (5/11) [11, 17, 18, 22, 24], the incidence of postoperative complications was lower in the GDFT group (OR 0.39, 95% CI 0.18–0.86; *P* = 0.02; *I* ^2^ = 77%). However, no statistically significant differences were found in the studies that used optimal SV as the goal (4/11) [4, 17, 21, 23] (Supplementary Fig. 1).

##### Study type and postoperative complications

**Fig. 7:**
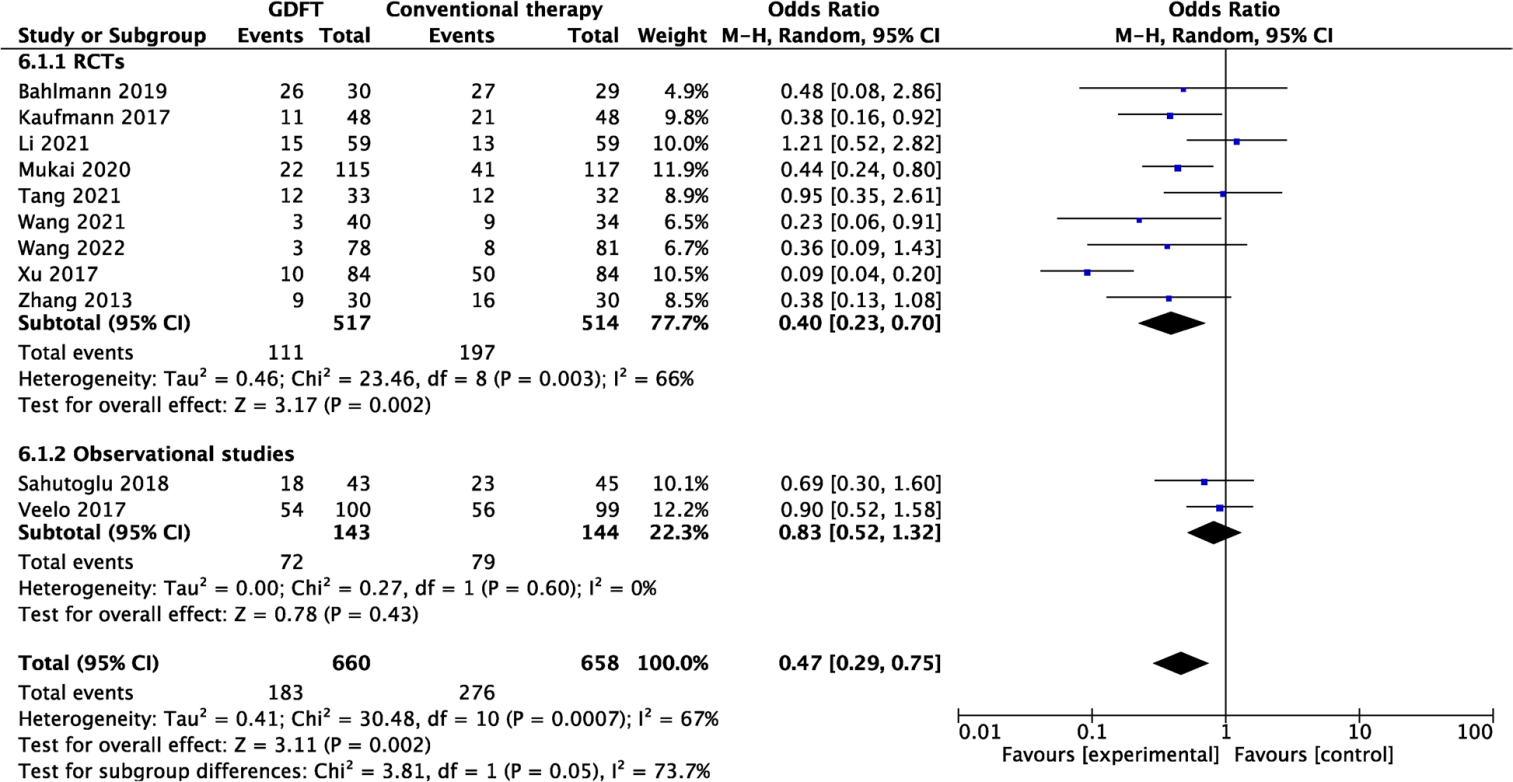
Forest plot comparing postoperative complications for the GDFT versus the conventional therapy according to study type

For RCTs (9/11) [4, 11, 16–18, 23–26], the incidence of postoperative complications was significantly lower in the GDFT group (OR 0.40, 95% CI 0.23–0.70; *P* = 0.002; *I* ^2^ = 66%). However, for observational studies, no significant difference in the incidence of postoperative complications between the two groups was found (2/11) [21, 22] (Fig. 7).

### Secondary outcomes

#### LOS

Ten studies reported the LOS during postoperative follow-up [4, 16–18, 21–26]. Four of these studies involved an esophagectomy [4, 18, 21, 24], and six involved lung resections [16, 17, 22, 23, 25, 26]. No statistically significant differences were noted for LOS (MD − 0.81, 95% CI − 1.65–0.02; *P* = 0.06; *I* ^2^ = 85%) (Supplementary Fig. 2).

#### Total fluid infusion

Ten studies reported the total fluid infusion [4, 11, 16–18, 21, 23–26]. Among these studies, the GDFT group was found to have a lower total fluid infusion (MD − 161.52, 95% CI − 318.92, − 4.12; *P* = 0.04; *I* ^2^ = 89%). As one of the included studies used RFT in the control group [26], we compared the total fluid infusion volume of GDFT compared with CFT separately. This analysis revealed similar results, as total fluid infusion in the GDFT group was lower than that in the CFT group (MD − 204.34, 95% CI − 353.09, − 55.59; *P* = 0.007; *I* ^2^ = 84%). According to the surgical procedure, the total fluid infusion was lower in the GDFT group for lung resections [11, 16, 17, 23, 25], while no significant difference was found for esophagectomies [4, 18, 21, 24] (Supplementary Fig. 3).

#### P/F ratio, inflammatory factor levels, and postoperative bowel function recovery time

Statistically significant differences were observed in the P/F ratio [11, 16, 24] (Supplementary Fig. 4) and inflammatory factors (IL-6, TNF-α) [16, 17, 25] (Supplementary Fig. 5) between the two groups. The GDFT group had 1.3 days shorter recovery time of bowel function [4, 17, 18] (Supplementary Fig. 6).

### Sensitivity analysis and publication bias

As we detected a moderate degree of heterogeneity in the primary outcome (*I* ^2^ = 67%), we performed a sensitivity analysis by omitting one study in turn to explore potential sources of heterogeneity. Excluding the study by Xu et al. [16] reduced the heterogeneity to 17% and increased the OR from 0.47 to 0.60. Excluding the study by Li et al. [26] reduced the heterogeneity to 65% and the OR from 0.47 to 0.42. Excluding both studies reduced the heterogeneity to 0% and increased the OR from 0.47 to 0.56.

Funnel plots were used to assess publication bias among the included studies. No evidence of publication bias for the primary outcome was suggested by visual inspection of the funnel plots (Supplementary Fig. 7).

## Discussion

Several previous meta-analyses found that the GDFT strategy reduced the mortality and complication rates in patients after abdominal surgery [7, 28, 29] as well as other non-cardiothoracic surgical procedures [15, 30]. However, none of these meta-analyses evaluated patients with OLV. OLV predisposes patients to interstitial fluid retention, which causes pulmonary edema through the combined effects of intrapulmonary shunting, hypoxic pulmonary vasoconstriction (HPV), ventilation/perfusion ratio mismatch, and collapse of the surgical-side lung [31, 32]. Thus, appropriate fluid management is equally important in OLV. Our meta-analysis is the first to evaluate the use of the GDFT approach in thoracic surgery with OLV.

Postoperative complications are significantly associated with perioperative fluid therapy [33]. The beneficial effect of GDFT on postoperative complications may be attributed to a few possible explanations. First, GDFT is associated with improved tissue perfusion and oxygenation. GDFT emphasizes “individualization” through advanced dynamic monitoring methods and effective standardized procedures to obtain optimal preload and oxygen delivery, thus improving the patient’s microcirculation and tissue oxygen supply [34]. Many previous studies have highlighted the importance of impaired tissue microcirculation and oxygenation in the pathogenesis of postoperative complications [35–37]. Our findings on the P/F ratio confirm this; however, only three studies were included in the P/F ratio analysis and we were unable to standardize the time point of monitoring among these studies [11, 16, 24]. Considering the effect time of HPV, we chose 30 min of OLV as the time point despite the fact that the results were highly heterogeneous (*I* ^2^ = 84%). This finding should thus be interpreted with caution, and more large studies on GDFT applied to OLV are needed to verify this. Second, the optimization of perioperative hemodynamics may have a beneficial effect on the systemic inflammatory response to surgery-related tissue damage, thereby reducing the incidence of postoperative complications [38, 39]. This is supported by our findings of reduced inflammatory response. Oxygenation and inflammatory response, both of which appear to be strongly correlated with a reduction in PPCs, in the GDFT group in our subgroup analysis for organ-specific complications, while favorable effects on postoperative renal and cardiovascular complications associated with preload and microcirculation were not confirmed. We speculate that insufficient intraoperative fluid infusion contributes more to AKI and heart failure [40], and our results showed that the difference in total fluid infusion between the GDFT and control groups was only − 161.52 mL (MD − 161.52; *P* = 0.04), which may not have been sufficient to affect tissue microcirculation. Secondly, the total fluid infusion in the CFT group was higher than that in the GDFT group, which may have resulted in some degree of fluid optimization, leading to increased cardiac output and thus reducing cardiac and renal complications [15].

The results of our subgroup analysis according to surgical procedure showed a more marked beneficial effect of GDFT in lung resection compared to esophagectomy. One potential explanation for this is that the mean operative time was 422 ± 98 min [4] for esophagectomy and 177.1 ± 57.6 min for lobectomy [26]. A longer operative time implies an increase in intraoperative fluid infusion, and as the results of our review shows, the maximum fluid infusion in the GDFT group for lung resection was 1384.7 ± 513.8 mL [25], while the minimum fluid infusion in the GDFT group for esophagectomy was 1999.2 ± 278.29 ml [21]. Fluid overload is associated with an increased incidence of postoperative complications [2, 41], and this is a point of concern. The current guidelines for thoracic surgery ERAS also recommend RFT (intraoperative and postoperative fluid infusion rate of 1–2 ml/kg/h, perioperative fluid balance < 1500 ml) [42]. This is because in lung resection surgery, OLV may lead to a decrease in pulmonary alveolar fluid clearance rate. Combined with the direct trauma of surgery, ischemia–reperfusion phenomena, and endothelial glycocalyx damage, the risks of interstitial edema, alveolar edema, and lung injury in patients increase [26, 40, 42]. Therefore, the purpose of RFT is to control fluid volume and minimize capillary hydrostatic pressure, preventing the risks mentioned above. Our study observed that the fluid volume in the GDFT group was lower than that in the control group (Supplementary Fig. 3). This indicates that GDFT can reduce unnecessary fluid administration, to some extent aligning with fluid restriction, thereby decreasing postoperative complications. Do anesthesiologists opt more for GDFT or the relatively fixed RFT mode recommended by the ERAS guidelines when making clinical decisions? We believe that currently, thoracic surgery fluid management strategy still adheres to a zero-balance approach [43], and GDFT’s superior predictive capacity for fluid responsiveness can better accommodate zero-balance. For thoracic surgery patients, the aim of GDFT is not only to decrease intraoperative fluid infusion but also to optimize end-organ perfusion with appropriate fluids based on dynamic parameters, thus stabilizing hemodynamic status [16]. Furthermore, patients with esophageal cancer are at risk for preoperative malnutrition [44]. In addition to selecting the optimal fluid resuscitation mode during surgery, it is also important to optimize perioperative fluid management by replacing intravenous fluids with the early resumption of oral fluids and diet [42]. Second, the hemodynamic goals of GDFT were not homogenous. In the studies on esophagectomy [4, 18, 21, 24], Bahlmann et al. [4] and Veelo et al. [21] used optimal SV as the hemodynamic goal, whereas Mukai et al. [18] used the optimal SVV. The GDFT strategy using the optimal SVV as the goal resulted in a reduction in the postoperative complications associated with esophagectomy [18, 24]. In our meta-analysis, the included studies primarily focused on two different GDFT strategies: optimizing SVV and optimizing SV. Some researchers argue that factors such as shunting caused by OLV, fluctuating intrathoracic pressures due to open-chest conditions, and compression of the heart and lungs by the surgical procedure can limit the predictive capability of SVV. This is because the changes in intrathoracic pressure and lung volume induced by positive pressure ventilation form the basis for alterations in SVV [4, 18]. Furthermore, during OLV, lung-protective ventilation with small tidal volumes (V_T_) is recommended. Renner et al. confirmed that SVV changes with varying V_T._ Specifically, when the V_T_ is excessively small (< 5 ml/kg), there is no significant change in SVV before and after volume expansion [45]. They are inclined to use SV as a goal for fluid therapy. However, SV optimization also has inherent limitations. Firstly, most studies included in our analysis used the Vigileo-FloTrac system to monitor hemodynamic parameters, which might underestimate SV due to differences between radial artery pressure measurements and central (femoral) measurements [4]. Additionally, when SV decreases by > 10%, its positive predictive value for fluid responsiveness is only 46% [46]. These findings underscore the risk of fluid overload associated with the SV optimization strategy, leading to postoperative complications. The potential concerns regarding SVV might be unnecessary. Firstly, Suehiro et al. demonstrated good predictability of SVV even in patients undergoing OLV (sensitivity: 82%, specificity: 92%) [47]. Fu et al. also indicated that SVV is a robust predictor of fluid responsiveness in OLV patients, with an area under the receiver operating characteristic curve (AUC) of 0.767 [48]. Additionally, even in cases of open thoracotomy, the ventilated (dependent) lung is not exposed to the atmosphere since its pleura remains intact, and the mediastinum isolates the lung from the atmosphere [47]. Therefore, if only one of SVV and SV can be selected, we believe SVV seems to provide a better prediction of fluid responsiveness during OLV, as confirmed by the results in “Hemodynamic goals and postoperative complications” section (Supplementary Fig. 1). However, for comprehensive optimization, we suggest combining dynamic indicators of fluid responsiveness (SVV) with other optimization parameters (such as SV, CI, and CO), which proves more effective than relying solely on either of them. There are various GDFT strategies available during surgery, particularly in the context of complex fluid management as seen in thoracic surgery. Implementing a sufficiently robust GDFT strategy is essential. In addition to the widely used options of SVV and SV, a recent study by Wang et al. [25] found that regional saturation of cerebral oxygenation (rScO_2_) monitoring can noninvasively show variations in the regional cerebral oxygen supply/demand balance in real-time and reduce POD [25] and thus may be a relevant GDFT goal for future clinical practice.

Moderate heterogeneity was noted for the pooled postoperative complications (primary outcome). We attribute this heterogeneity primarily to the inclusion of two non-randomized studies of the effects of interventions (NRSI) in the analysis of the primary outcome [21, 22]. The Cochrane Handbook explicitly states that authors should consider the potential for increased heterogeneity due to confounding factors and bias in NRSI [49]. Firstly, both studies could not randomize participants like RCTs, leading to differences in baseline characteristics among different groups. Non-randomization increased the likelihood of selection bias in these two studies. The study by Veelo et al. [21] utilized a before-after study design. The passage of time could influence surgical techniques and experience. Additionally, there were statistically significant differences between the GDFT and CFT groups in baseline characteristics such as surgical technique and the amount of epidural analgesia used. These factors increased the risk of selection bias. While Veelo et al. attempted to adjust for these confounding effects in their multivariate analysis, the inherent limitations of non-randomization still persist. The study by Sahutoglu et al. [22] is retrospective. We are uncertain about the factors that influenced group allocation, as the authors only mention that patient data was obtained through screening of the patient files. They did not specify whether propensity score matching was used to adjust for differences in baseline characteristics between the groups. While they reported that several baseline characteristics did not show statistically significant differences, their assessment of individual baseline characteristics appears limited. Some factors that could influence the outcomes, such as surgical approach (thoracotomy/thoracoscopic), surgery duration, and pre-existing comorbidities, were not mentioned. These omissions could introduce a risk of selection bias. When selection bias leads to an imbalance in prognostic factors between the GDFT and CFT groups, confounding occurs, which can have two effects: (1) altering the estimation of intervention effects and (2) introducing excessive heterogeneity into the study [49]. We believe this is the primary reason for the moderate heterogeneity in our study results and the inconsistency between the subgroup analysis (based on study type, Fig. 7) results. Secondly, we are uncertain whether the two studies employed blinding in outcome assessment, which could introduce bias in the measurement of outcomes. Lastly, in Veelo et al.’s study [21], patients in the CFT group received more epidural anesthesia, and those with epidural anesthesia had poorer postoperative mobility (to avoid catheter displacement). This further influenced the outcomes, increasing the risk of pulmonary infections. Additionally, it also introduced bias due to deviations from intended interventions in the study. The aforementioned two NRSIs exhibit non-randomization, bias, and between-group differences in individual baseline characteristics. We infer that these factors could be the primary contributors to the moderate heterogeneity observed in the primary outcome. However, the overall risk of bias in both studies was assessed as low (Fig. 3). Veelo et al. also employed specific adjustment techniques to mitigate the impact of the aforementioned confounding (multivariable analysis) [21]. Furthermore, excluding the two NRSIs did not obviously alter the effect size of the results (3.11 VS 3.17). Therefore, we ultimately decided to include them in the pool of results. We also conducted a sensitivity analysis and found a reduction in the heterogeneity to 0% after excluding the studies conducted by Li et al. [26] and Xu et al. [16]. In the study by Li et al., the fluid therapy used for the control group was RFT [26], which may have had some impact on the heterogeneity of our results. In the study conducted by Xu et al., [16] instead of reporting the total number of postoperative complications, the authors reported only the number and rate of individual complications. As we were unable to request additional original study data from the corresponding authors, we simply added up the number of each complication. This may have ultimately led to a high reported rate of complications and resulted in heterogeneity.

Our study has several limitations. First, The included patients in the meta-analysis come from different hospitals, where caseload and medical standards may vary. Therefore, outcomes such as complications, short-term mortality, and hospital stay might not necessarily reflect the actual clinical impact of the intervention on patient outcomes. The observed “benefits” in these data may be confined to statistical significance. This characteristic is particularly evident in short-term mortality, where the short-term mortality rate for esophageal cancer depends on the caseload of the treating hospital [50], rather than the implementation of GDFT. Hence, although five included studies reported short-term mortality, we did not analyze it. Second, Sivakumar et al. found that meta-analysis was several times more likely to find a significant treatment effect than subsequent large RCTs. There was a strong tendency towards positive findings in meta-analysis not substantiated by subsequent large RCTs. Furthermore, the heterogeneity and publication bias in the meta-analysis can increase the risk of type 1 errors, leading to the potential discovery of treatment effects that may not be truly accurate [51]. These are inherent limitations of meta-analysis. As for whether GDFT can genuinely impact the occurrence of postoperative complications and even long-term clinical outcomes after thoracic surgery with OLV, further large-scale RCTs are needed for validation. Therefore, our study findings should be interpreted cautiously. Third, the definitions of postoperative complications used in the studies were not homogenous, and thus, the severity of the complications reported may have been inconsistent. We were unable to homogenize the definitions of postoperative complications in the original studies. Fourth, as previously mentioned, the potential biases and confounding factors present in the two included observational studies may have a potential impact on the results. Finally, as discussed above, there is a large discrepancy in the duration of the surgery according to surgical procedure. The trauma and other effects that a longer duration of surgery can have on the patients may lead to a different risk–benefit balance according to the procedure. We attempted to identify cutoff values for the correlation between procedure length and complications; however, the lack of data prevented us from conducting further studies and we could only perform subgroup analyses to explore potential factors.

## Conclusion

Perioperative GDFT reduces the incidence of postoperative complications, particularly PPCs and anastomotic leakage. The GDFT strategy has a positive effect on reducing postoperative complications in lung resections, whereas this effect is not clearly evident in esophagectomy. In addition, GDFT reduces postoperative mortality, decreases total fluid infusion, improves the oxygenation index, and shortens the time to recovery of bowel function. However, GDFT has no effect on LOS, postoperative cardiovascular or renal complications, or POD. GDFT strategies using the SVV and/or CI as goals have been associated with better outcomes.

### Supplementary Information

**Additional file 1: Fig. S1.** Forest plot comparing postoperative complications for the GDFT versus CFT group according to hemodynamic goals. **Fig. S2.** Forest plot comparing the LOS of the GDFT versus CFT group. **Fig. S3.** Forest plot comparing total fluid infusion for the GDFT versus the control group according to surgical procedure. **Fig. S4.** Forest plot comparing the PaO2/FiO2 ratio for the GDFT versus CFT group. **Fig. S5.** Forest plot comparing the inflammatory factors (TNF-α, Il-6) for the GDFT versus the CFT group. **Fig. S6.** Forest plot comparing the time to recovery of bowel function for the GDFT versus the CFT group. **Fig. S7.** Funnel plot for meta-analysis of the postoperative complications. **Table 1.** Individual search strategies for all the databases and the number of search results.

**Additional file 2. **PRISMA Checklist.

## Data Availability

The original contributions presented in the study are included in the article/supplementary material, and further inquiries can be directed to the corresponding author/s.

## Abbreviations

GDFT: Goal-directed fluid therapy
OLV: One-lung ventilation
SVV: Stroke volume variation
SV: Stroke volume
CVP: Central venous pressure
CI: Cardiac index
MAP: Mean arterial pressure
rScO_2_: Regional saturation of cerebral oxygenation
BP: Blood pressure
PPCs: Postoperative pulmonary complications
AKI: Acute kidney injury
POD: Postoperative delirium
LOS: Length of hospital stay
RCTs: Randomized controlled trials
V_T_: Tidal volume
OR: Odds ratio
MD: Mean difference
CI: Confidence interval
ROBINS-I: Risk of Bias In Non-randomized Study of Interventions
NRSI: Non-randomized studies of the effects of interventions
BMI: Body mass index
ASA: American Society of Anesthesiologists
CFT: Conventional fluid therapy
RFT: Restrictive fluid therapy
MeSH: Medical subject terms
HPV: Hypoxic pulmonary vasoconstriction
