# Supplementary material for: Effect of perioperative goal-directed fluid therapy on postoperative complications after thoracic surgery with one-lung ventilation: a systematic review and meta-analysis
Source: World J Surg Oncol. 2023 Sep 18;21:297. doi: 10.1186/s12957-023-03169-5 (PMC10506328; doi:10.1186/s12957-023-03169-5)
Supplement: Supplementary file 1 — Additional file 1: Fig. S1. Forest plot comparing postoperative complications for the GDFT versus CFT group according to hemodynamic goals. Fig. S2. Forest plot comparing the LOS of the GDFT versus CFT group. Fig. S3. Forest plot comparing total fluid infusion for the GDFT versus the control group according to surgical procedure. Fig. S4. Forest plot comparing the PaO2/FiO2 ratio for the GDFT versus CFT group. Fig. S5. Forest plot comparing the inflammatory factors (TNF-α, Il-6) for the GDFT versus the CFT group. Fig. S6. Forest plot comparing the time to recovery of bowel function for the GDFT versus the CFT group. Fig. S7. Funnel plot for meta-analysis of the postoperative complications. Table 1. Individual search strategies for all the databases and the number of search results. [file 12957_2023_3169_MOESM1_ESM.doc]

**Supplementary materials**


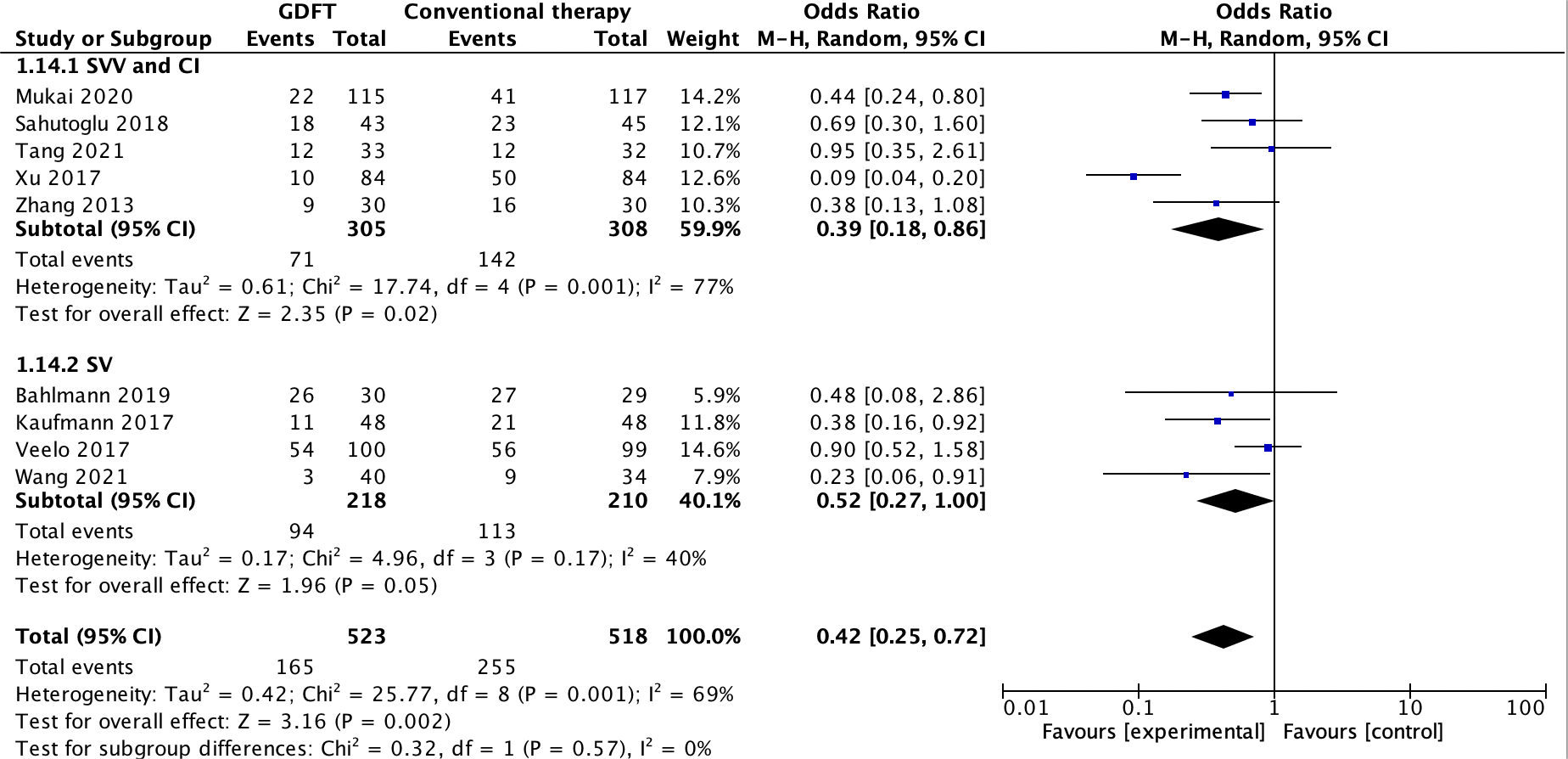


Supplementary Figure 1: Forest plot comparing postoperative complications for the GDFT versus CFT group according to hemodynamic goals.


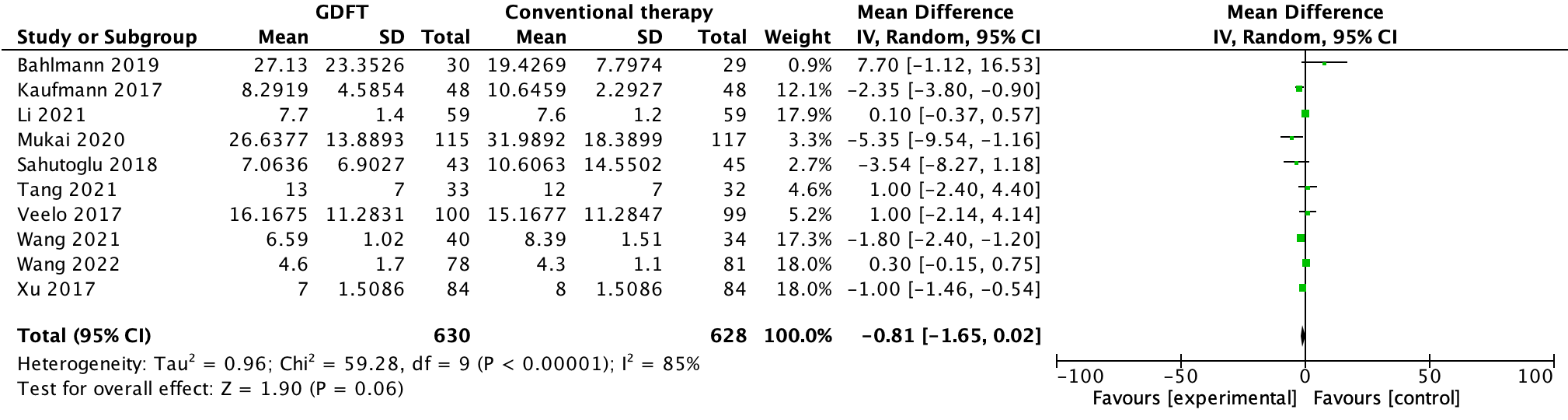


Supplementary Figure 2: Forest plot comparing the LOS of the GDFT versus CFT group.


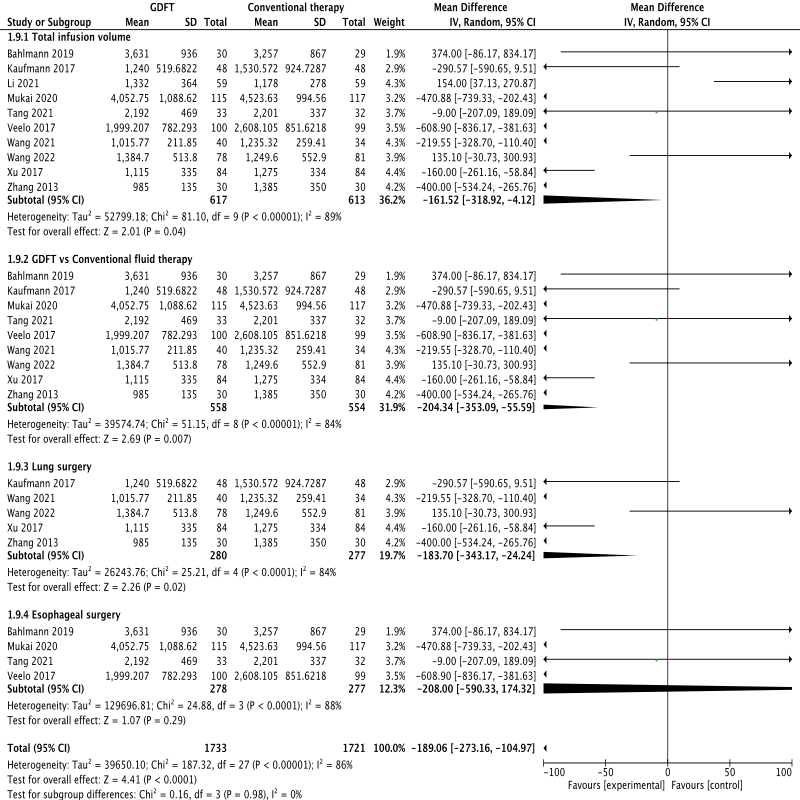


Supplementary Figure 3: Forest plot comparing total fluid infusion for the GDFT versus control group according to surgical procedure.


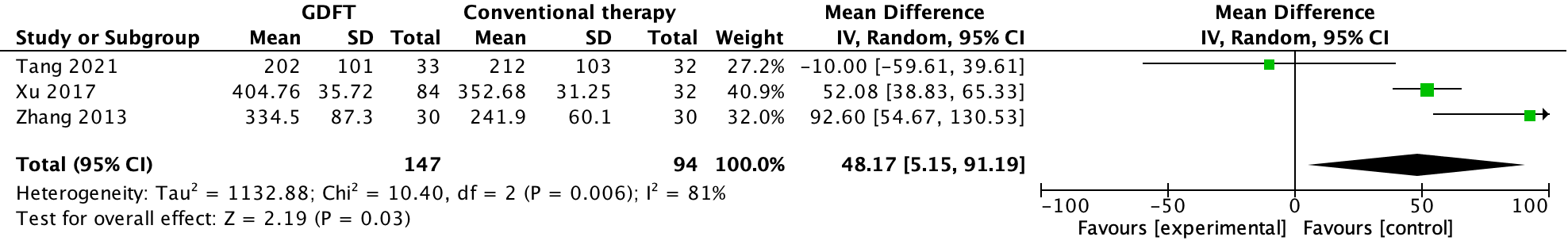


Supplementary Figure 4: Forest plot comparing the PaO_2_/FiO_2_ ratio for the GDFT versus CFT group.


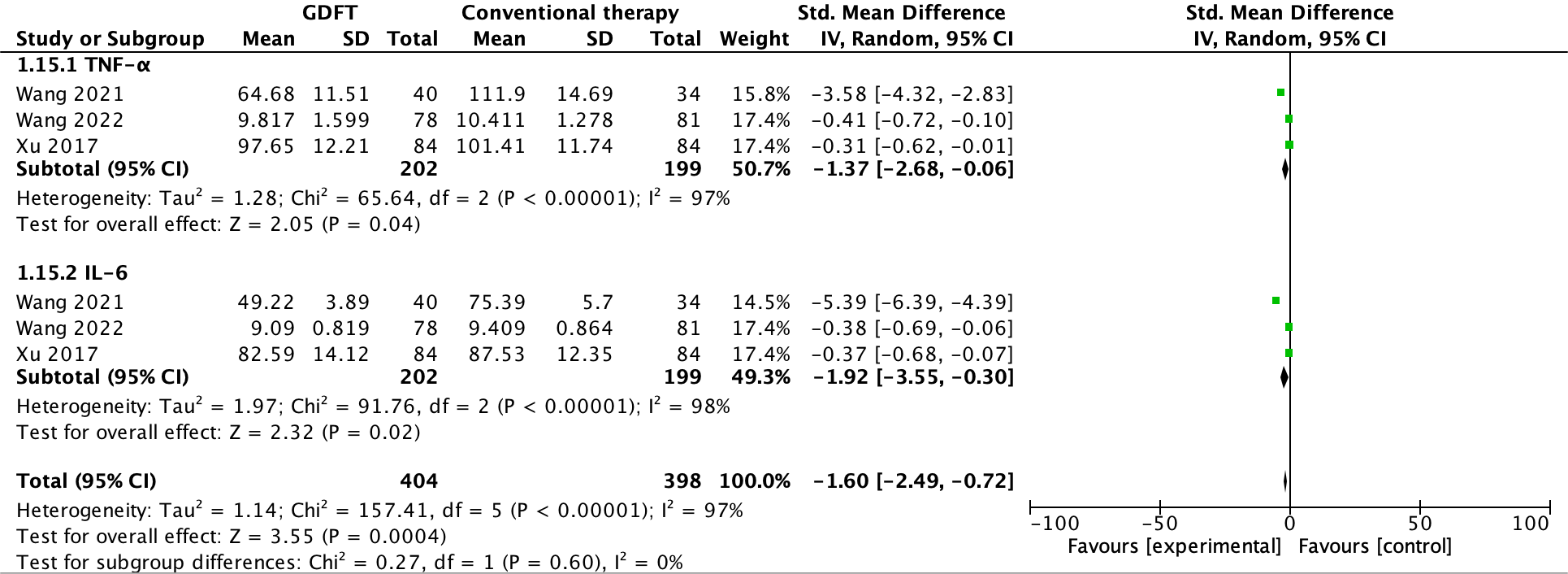


Supplementary Figure 5: Forest plot comparing the inflammatory factors (TNF-α, Il-6) for the GDFT versus the CFT group.


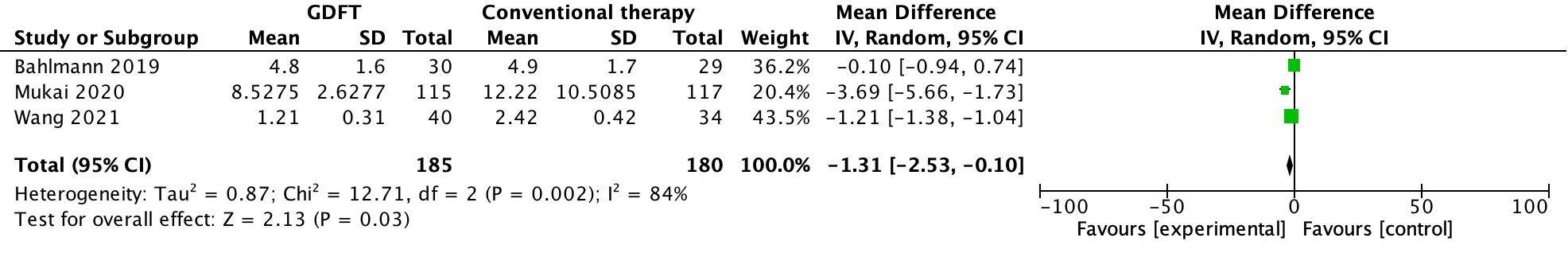


Supplementary Figure 6: Forest plot comparing the time to recovery of bowel function for the GDFT versus the CFT group.


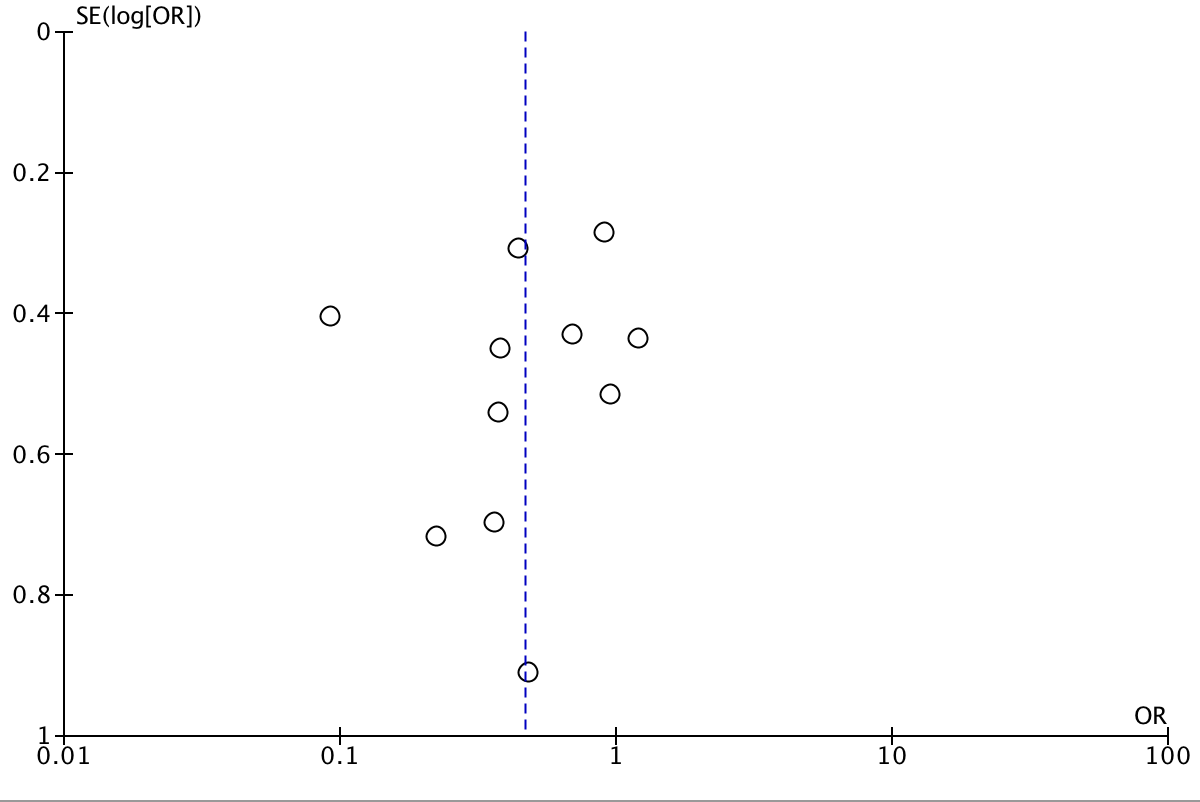


Supplementary Figure 7: Funnel plot for meta-analysis of the postoperative complications.

Supplementary Table 1: Individual search strategies for all the databases and the number of search results.

| Database | Search Strategy | Result |
| --- | --- | --- |
| MEDLINE via PubMed | ((((((((((((goal-directed fluid therapy) OR (goal directed fluid therapy)) OR (goal-directed therapy)) OR (goal directed therapy)) OR (goal-directed haemodynamic therapy)) OR (goal directed haemodynamic therapy)) OR (GDT)) OR (GDHT)) OR (GDFT)) OR (goal-directed fluid)) OR (goal-oriented fluid therapy)) OR (goal-oriented therapy)) AND ((((((((((((((((((((one-lung ventilation) OR (one lung ventilation)) OR (single-lung ventilation)) OR (single lung ventilation)) OR (OLV)) OR (thoracic surgery)) OR (lung surgery)) OR (esophageal surgery)) OR (pneumonectomy)) OR (pulmonectomy)) OR (pneumectomy)) OR (lobectomy)) OR (wedge resection)) OR (lung resection)) OR (lung resection surgery)) OR (lung cancer)) OR (lung cancer surgery)) OR (esophagectomy)) OR (oesophagectomy)) OR (esophageal)) | 938 |
| Cochrane | #1: (goal-directed fluid therapy) OR (goal directed fluid therapy) OR (goal-directed therapy) OR (goal directed therapy) OR (goal-directed haemodynamic therapy)#2: (goal directed haemodynamic therapy) OR (GDT) OR (GDHT) OR (GDFT) OR (goal-directed fluid)#3: (goal-oriented fluid therapy) OR (goal-oriented therapy) #4: #1 or #2 or #3  #5: (one-lung ventilation) OR (one lung ventilation) OR (single-lung ventilation) OR (single lung ventilation) OR (OLV)  #6: (thoracic surgery) OR (lung surgery) OR (esophageal surgery) OR (pneumonectomy) OR (pulmonectomy)  #7: (pneumectomy) OR (lobectomy) OR (wedge resection) OR (lung resection) OR (lung resection surgery)  #8: (lung cancer) OR (lung cancer surgery) OR (esophagectomy) OR (oesophagectomy) OR (esophageal)  #9: #5 or #6 or #7 or #8  #10: #4 and #9 | 778 |
| Embase | #1: 'goal-directed fluid therapy':ab,ti OR 'goal directed fluid therapy':ab,ti OR 'goal-directed therapy':ab,ti OR 'goal directed therapy':ab,ti OR 'goal-directed haemodynamic therapy':ab,ti OR 'goal directed haemodynamic therapy':ab,ti OR 'gdt':ab,ti OR 'gdht':ab,ti OR 'gdft':ab,ti OR 'goal-directed fluid':ab,ti OR 'goal-oriented fluid therapy':ab,ti OR 'goal-oriented therapy':ab,ti  #2: 'one-lung ventilation':ab,ti OR 'one lung ventilation':ab,ti OR 'single-lung ventilation':ab,ti OR 'single lung ventilation':ab,ti OR 'olv':ab,ti OR 'thoracic surgery':ab,ti OR 'lung surgery':ab,ti OR 'esophageal surgery':ab,ti OR 'pneumonectomy':ab,ti OR 'pulmonectomy':ab,ti OR 'pneumectomy':ab,ti OR 'lobectomy':ab,ti OR 'wedge resection':ab,ti OR 'lung resection':ab,ti OR 'lung resection surgery':ab,ti OR 'lung cancer':ab,ti OR 'lung cancer surgery':ab,ti OR 'esophagectomy':ab,ti OR 'oesophagectomy':ab,ti OR 'esophageal':ab,ti  #3: #1 AND #2 | 99 |
| Web of Science | **#1: TS=("**goal-directed fluid therapy**" OR "**goal directed fluid therapy**" OR "**goal-directed therapy**" OR "**goal directed therapy**" OR "**goal-directed haemodynamic therapy**" OR "**goal directed haemodynamic therapy**" OR "**GDT**" OR "**GDHT**" OR "**GDFT**" OR "**goal-directed fluid**" OR "**goal-oriented fluid therapy**" OR "**goal-oriented therapy**")**  **#2: TS=("**one-lung ventilation**" OR "**one lung ventilation**" OR "**single-lung ventilation**" OR "**single lung ventilation**" OR "**OLV**" OR "**thoracic surgery**" OR "**lung surgery**" OR "**esophageal surgery**" OR "**pneumonectomy**" OR "**pulmonectomy**" OR "**pneumectomy**" OR "**lobectomy**" OR "**wedge resection**" OR "**lung resection**" OR "**lung resection surgery**" OR "**lung cancer**" OR "**lung cancer surgery**" OR "**esophagectomy**" OR "**oesophagectomy**" OR "**esophageal**")**  **#3: #1 AND #2** | 121 |

.
