# Supplementary material for: Effect of perioperative goal-directed fluid therapy on postoperative complications after thoracic surgery with one-lung ventilation: a systematic review and meta-analysis
Source: World J Surg Oncol. 2023 Sep 18;21:297. doi: 10.1186/s12957-023-03169-5 (PMC10506328; doi:10.1186/s12957-023-03169-5)
Supplement: Supplementary file 2 — Additional file 2. PRISMA Checklist. [file 12957_2023_3169_MOESM2_ESM.doc]

| **Section and Topic** | **Item #** | **Checklist item** | **Location where item is reported** |
| --- | --- | --- | --- |
| **TITLE** | | |  |
| Title | 1 | Identify the report as a systematic review. | The report is identified as a systematic review and meta-analysis in page 1. (**Title**) |
| **ABSTRACT** | | |  |
| Abstract | 2 | See the PRISMA 2020 for Abstracts checklist. | the structured abstract includes Objective, Research Design and Methods, Results, and Conclusion in page 1. (**Abstract**) |
| **INTRODUCTION** | | |  |
| Rationale | 3 | Describe the rationale for the review in the context of existing knowledge. | Page 1-2 (**Introduction**)；Paragraphs 1-2 |
| Objectives | 4 | Provide an explicit statement of the objective(s) or question(s) the review addresses. | Page 1-2 (**Introduction**)；Paragraph 3 |
| **METHODS** | | |  |
| Eligibility criteria | 5 | Specify the inclusion and exclusion criteria for the review and how studies were grouped for the syntheses. | Page 2 （**Methods; Inclusion and Exclusion Criteria**） |
| Information sources | 6 | Specify all databases, registers, websites, organisations, reference lists and other sources searched or consulted to identify studies. Specify the date when each source was last searched or consulted. | Page 2（**Methods；Search Strategy**） |
| Search strategy | 7 | Present the full search strategies for all databases, registers and websites, including any filters and limits used. | Page 2 and Page 4-5（**Method；Search Strategy；supplementary table 1**） |
| Selection process | 8 | Specify the methods used to decide whether a study met the inclusion criteria of the review, including how many reviewers screened each record and each report retrieved, whether they worked independently, and if applicable, details of automation tools used in the process. | Page 3**（Results; Selection of studies and study characteristics）** |
| Data collection process | 9 | Specify the methods used to collect data from reports, including how many reviewers collected data from each report, whether they worked independently, any processes for obtaining or confirming data from study investigators, and if applicable, details of automation tools used in the process. | Page 3（**Data extraction and Quality assessment）** |
| Data items | 10a | List and define all outcomes for which data were sought. Specify whether all results that were compatible with each outcome domain in each study were sought (e.g. for all measures, time points, analyses), and if not, the methods used to decide which results to collect. | Page 2-3**（Methods; Outcome Measures）** |
|  | 10b | List and define all other variables for which data were sought (e.g. participant and intervention characteristics, funding sources). Describe any assumptions made about any missing or unclear information. | Page 3（**Data extraction and Quality assessment）** |
| Study risk of bias assessment | 11 | Specify the methods used to assess risk of bias in the included studies, including details of the tool(s) used, how many reviewers assessed each study and whether they worked independently, and if applicable, details of automation tools used in the process. | Page 3（**Data extraction and Quality assessment）** |
| Effect measures | 12 | Specify for each outcome the effect measure(s) (e.g. risk ratio, mean difference) used in the synthesis or presentation of results. | Page 3**（Methods; Statistical Analysis）** |
| Synthesis methods | 13a | Describe the processes used to decide which studies were eligible for each synthesis (e.g. tabulating the study intervention characteristics and comparing against the planned groups for each synthesis (item #5)). | Page 3**（Results; Selection of studies and study characteristics）** |
|  | 13b | Describe any methods required to prepare the data for presentation or synthesis, such as handling of missing summary statistics, or data conversions. | Page 3**（Methods; Statistical Analysis）** |
|  | 13c | Describe any methods used to tabulate or visually display results of individual studies and syntheses. | Page 3（**Statistical Analysis；Selection of studies and study characteristics）** |
|  | 13d | Describe any methods used to synthesize results and provide a rationale for the choice(s). If meta-analysis was performed, describe the model(s), method(s) to identify the presence and extent of statistical heterogeneity, and software package(s) used. | Page 3**（Methods; Statistical Analysis）** |
|  | 13e | Describe any methods used to explore possible causes of heterogeneity among study results (e.g. subgroup analysis, meta-regression). | Page 6**（Results; Sensitivity Analysis and Publication Bias）** |
|  | 13f | Describe any sensitivity analyses conducted to assess robustness of the synthesized results. | Page 6**（Results; Sensitivity Analysis and Publication Bias）** |
| Reporting bias assessment | 14 | Describe any methods used to assess risk of bias due to missing results in a synthesis (arising from reporting biases). | Page 6（**Results; Sensitivity Analysis and Publication Bias）** |
| Certainty assessment | 15 | Describe any methods used to assess certainty (or confidence) in the body of evidence for an outcome. | Page 3**（Methods; Statistical Analysis）** |
| **RESULTS** | | |  |
| Study selection | 16a | Describe the results of the search and selection process, from the number of records identified in the search to the number of studies included in the review, ideally using a flow diagram. | Page 3 and Page 5**（Results; Selection of studies and study characteristics; Figure 1）** |
|  | 16b | Cite studies that might appear to meet the inclusion criteria, but which were excluded, and explain why they were excluded. | Page 3 and Page 5**（Results; Selection of studies and study characteristics; Figure 1）** |
| Study characteristics | 17 | Cite each included study and present its characteristics. | Page 3 and Page 5**（Results; Selection of studies and study characteristics; Figure 1）** |
| Risk of bias in studies | 18 | Present assessments of risk of bias for each included study. | Page 3（**Quality assessment; Figure 2 and 3）** |
| Results of individual studies | 19 | For all outcomes, present, for each study: (a) summary statistics for each group (where appropriate) and (b) an effect estimate and its precision (e.g. confidence/credible interval), ideally using structured tables or plots. | **Result; Figure 4-7; supplementary figure 1-7** |
| Results of syntheses | 20a | For each synthesis, briefly summarise the characteristics and risk of bias among contributing studies. | Page 3 **(Results; Quality assessment; Figure 2 and 3)** |
|  | 20b | Present results of all statistical syntheses conducted. If meta-analysis was done, present for each the summary estimate and its precision (e.g. confidence/credible interval) and measures of statistical heterogeneity. If comparing groups, describe the direction of the effect. | **Result; Figure 4-7; supplementary figure 1-7** |
|  | 20c | Present results of all investigations of possible causes of heterogeneity among study results. | Page 6 **(Results; Sensitivity Analysis and Publication Bias);** Page 11 **(Discussion section; Paragraph 4)** |
|  | 20d | Present results of all sensitivity analyses conducted to assess the robustness of the synthesized results. | Page 6 **(Results; Sensitivity Analysis and Publication Bias);** Page 11 **(Discussion section; Paragraph 4)** |
| Reporting biases | 21 | Present assessments of risk of bias due to missing results (arising from reporting biases) for each synthesis assessed. | Page 6 **(Results; Sensitivity Analysis and Publication Bias; supplementary figure 7)** |
| Certainty of evidence | 22 | Present assessments of certainty (or confidence) in the body of evidence for each outcome assessed. | **Result; Figure 4-7; supplementary figure 1-7** |
| **DISCUSSION** | | |  |
| Discussion | 23a | Provide a general interpretation of the results in the context of other evidence. | Page 6-11 (**Discussion Section: Paragraphs 1-3**） |
|  | 23b | Discuss any limitations of the evidence included in the review. | Page 11-12（**Discussion Section: Paragraph 4-5）** |
|  | 23c | Discuss any limitations of the review processes used. | Page 11-12（**Discussion Section: Paragraph 5）** |
|  | 23d | Discuss implications of the results for practice, policy, and future research. | Page 6-12 (**Discussion Section: Paragraphs 1-5**） |
| **OTHER INFORMATION** | | |  |
| Registration and protocol | 24a | Provide registration information for the review, including register name and registration number, or state that the review was not registered. | Page 2（ **Methods; Systematic Review Registration**）Page 2**（Methods; Search Strategy）** |
|  | 24b | Indicate where the review protocol can be accessed, or state that a protocol was not prepared. | Page 2**（Methods; Search Strategy）** |
|  | 24c | Describe and explain any amendments to information provided at registration or in the protocol. | Page 12（**Discussion Section: Paragraph 5）** |
| Support | 25 | Describe sources of financial or non-financial support for the review, and the role of the funders or sponsors in the review. | Page 12 (**Acknowledgement)** |
| Competing interests | 26 | Declare any competing interests of review authors. | Page 13 **( Competing interests)** |
| Availability of data, code and other materials | 27 | Report which of the following are publicly available and where they can be found: template data collection forms; data extracted from included studies; data used for all analyses; analytic code; any other materials used in the review. | Page 12 **(Availability of data and materials)** |

*From:*  Page MJ, McKenzie JE, Bossuyt PM, Boutron I, Hoffmann TC, Mulrow CD, et al. The PRISMA 2020 statement: an updated guideline for reporting systematic reviews. BMJ 2021;372:n71. doi: 10.1136/bmj.n71

For more information, visit: <http://www.prisma-statement.org/>
